# Identifying elderly patients at risk of readmission after discharge from a short-stay unit in the emergency department using performance-based tests of daily activities

**DOI:** 10.1186/s12877-020-01591-y

**Published:** 2020-06-22

**Authors:** Louise Moeldrup Nielsen, Thomas Maribo, Hans Kirkegaard, Mette Kops Bjerregaard, Lisa Gregersen Oestergaard

**Affiliations:** 1 Department of Physiotherapy and Occupational Therapy, Aarhus University Hospital, Arhus, Denmark; 2 Department of Occupational Therapy, VIA University College, Aarhus, Denmark; 3 Department of Public Health, Aarhus University, Aarhus, Denmark; 4 DEFACTUM, Central Denmark Region, Aarhus, Denmark; 5 Research Centre for Emergency Medicine, Aarhus University Hospital and Aarhus University, Aarhus, Denmark; 6 Neurorehabilitation and Research Centre, Hammel, Denmark; 7 The Research Initiative for Activity Studies and Occupational Therapy, General Practice, Department of Public Health, University of Southern Denmark, Odense, Denmark

**Keywords:** Activity limitations, Rehabilitation, Readmission, Elderly patients, Emergency department, Performance-based, Measurement

## Abstract

**Background:**

Readmission is a serious and adverse event for elderly patients. Despite efforts, predicting the risk of readmission remains imprecise. The objective of this study is to examine if performance-based tests of daily activities can identify elderly patients at risk of readmission within 26 weeks after discharge from a short-stay unit in the emergency department.

**Methods:**

The current study is an observational study based on data from 144 elderly patients included in a previous non-randomised controlled trial. Before discharge, patients were assessed for limitations in performing daily activities using three performance-based tests with predetermined cut-off values: the Assessment of Motor and Process Skills, Timed Up and Go and the 30s-Chair Stand Test. Outcome was risk of readmission within 26 weeks after discharge.

**Results:**

Limitations in performing daily activities were associated with risk of readmission as measured by the Assessment of Motor and Process Skills motor scale (Crude OR = 4.38 [1.36; 14.12]), (Adjusted OR = 4.17 [1.18; 14.75]) and the 30s-Chair Stand Test (Adjusted OR = 3.36 [1.42; 7.93]). No significant associations were found in regards to other measures.

**Conclusion:**

The Assessment of Motor and Process Skills motor scale and the age, gender and comorbidity adjusted 30s-Chair Stand Test can identify elderly patients at increased risk of readmission after discharge from the emergency department. The results were limited by one-third of the patients did not perform the Assessment of Motor and Process Skills and the association between 30s-Chair Stand Test and risk of readmission were only positive when adjusted for age, gender and comorbidity.

## Background

The number of elderly patients (aged 65 or above) admitted to emergency departments (EDs) has increased in recent decades, and elderly patients account for up to 25% of all ED visits [1, 2]. Readmission is a serious and adverse event for elderly patients [1]. Approximately 10–20% of the elderly patients who visit the ED are readmitted within 30 days after discharge [3, 4], while between 30 and 40% are readmitted within 26 weeks [5, 6]. Readmission has consequences for both the patient and for society. For the patient, readmission disrupts their daily routines and exposes them to hospitalisation-related complications and infections [2, 7, 8]. In addition, during hospitalisation, the patient is more likely to develop hospitalisation-associated disability and confusion [9]. The societal impact of readmission is that it consumes resources and subsequently leads to increased health care costs [8].

One strategy to reduce readmissions is the use of risk stratification to identify patients who are at increased risk of readmission [4, 10]. For that purpose, predictive models for readmission have been developed based on socio-demographic and clinical variables, and some of those models are validated in different populations such as medical and/or surgical patients in both acute and general hospital settings [4, 10–12]. The LACE index (Length of stay, Acuity of admission, Charlson comorbidity index and Emergency department visits in the past 6 months) was developed as a simple prediction index based on variables from the health care system [11]. It has been tested in different countries but has shown poor performance, which raises questions regarding its clinical utility [11]. A systematic review from 2011 including 26 models for prediction of risk of readmission concluded that the identified models have limited predictive ability and the majority of the models build on factors such as diagnosis or comorbidity, age, gender and previous use of medical services [10]. The Dynamic Silver Code (DSC) based on administrative data, could in a validation study identify elderly patients at risk of readmission and mortality when admitted to the ED. However, further studies are needed to validate DSC in other settings [13].

Despite efforts to develop models to identify patients at risk of readmission, predicting the risk of readmission remains imprecise, and some studies suggest that unmeasured patient-related factors, such as limitations in performing daily activities, may be related to this [12, 14].

The significance of measurement of limitations in performing daily activities on risk of readmission in elderly patients has not yet been extensively explored. Some studies have included tests with self-reported limitations in performing daily activities as a risk factor for readmission [4, 15, 16], although the use of self-reported data in this population poses some challenges. Self-reported data provides important information on previous ability to perform daily activities; however, elderly patients often overestimate their abilities [17]. This may affect the value of the information [14, 17]. Other studies have included performance-based tests of limitations in performing daily activities in relation to basic mobility such as pace count, grip strength, Timed Up and GO (TUG) and the Short Physical Performance Battery [14, 18, 19]. However, the use of such tests has yielded divergent results [14, 18, 19], and there is a lack of knowledge about whether tests of basic mobility are able to identify elderly patients at risk of readmission after discharge from a short-stay unit in the ED. In addition, little is known about the use of more comprehensive performance-based tests measuring quality in the performance of daily activities in areas other than mobility. Hence, the objective of this study was to examine if performance-based tests of daily activities can identify elderly patients at risk of readmission after discharge from a short-stay unit in the ED.

## Methods

### Design, setting and participants

The current study is an observational study based on data from 144 elderly patients included in a previous non-randomised controlled trial [20]. The objective of the non-randomised controlled trial was to examine the effectiveness of an intervention aimed at reducing the risk of readmission in elderly patients discharged from a short-stay unit in the ED. A total of 375 patients were included from March to December 2014. Inclusion criteria were age 65 and over with a medical diagnosis and admitted on weekdays to a short-stay unit in the ED at a university hospital in Denmark. Patients admitted from a nursing home, patients transferred to other hospital departments, patients unable to communicate in Danish and patients declared terminally ill were excluded. The intervention in the previous non-randomised controlled trial consisted of an assessment of elderly patients’ limitations in performing daily activities using three performance-based tests, referral to further rehabilitation in primary care and a follow-up visit at home the day after discharge. The intervention was not effective in reducing the risk of readmission as there were no tendency towards differences between the intervention and control groups [20]. In the present study, we use data from patients in the intervention group to examine the association between limitations in performing daily activities measured at baseline using three different performance-based tests and the risk of readmission within 26 weeks after discharge.

### Procedures

Patients who met the inclusion criteria were recruited on weekday mornings prior to their discharge. All included patients gave written consent for their enrolment. An occupational therapist performed the Assessment of Motor and Process Skills (AMPS) as the first of the three tests [21, 22]. Subsequently, a physiotherapist performed the 30s-Chair Stand test (30s-CST) [23, 24] and Timed Up and Go (TUG) [25]. The occupational therapist and physiotherapist participated in a two-week training period where they received supervision and feedback to ensure the correct implementation of the assessments.

### Outcome

The primary outcome was all-cause readmission to hospital within 26 weeks after discharge from a short-stay unit in the ED. Data on readmission were obtained from the Danish National Patient Registry, which allowed for complete follow-up data on all the participants.

### Tests

#### Assessment of motor and process skills (AMPS)

The AMPS is an observational assessment that measures the performance quality of tasks related to daily activities in a natural, task-relevant environment [21, 22]. Quality of performance is determined by the patient’s effort, efficiency, safety, and independence with goal-directed task skills. Timeframe for administer the AMPS is approximately 45 min [21].

The AMPS was administered in three phases. First, the occupational therapist interviewed the patient with the purpose of obtaining information about the patient’s daily activities in order to select two tasks of relevance to the patient to perform. Then the occupational therapist observed the patient’s performance of these two tasks and evaluated the quality of the performance. In the AMPS evaluation, the patient’s performance is scored on two scales: one measuring motor skills and one measuring process skills. Limitation in performing daily activities was determined using the following cut-off values: AMPS motor ability < 1.50 logits and process ability < 1.00 logits [21].

#### 30s-chair stand test (30s-CST)

The 30s-CST assesses lower body strength as an important proxy for mobility [23]. Timeframe for administer the 30s-CST is approximately 5 min. The 30s-CST was administered using a chair with a seat height of 43 cm. When given the signal to “go”, the participant rose to a full standing position and was instructed to complete as many full stands as possible within the 30-s time limit. A cut-off value defined as 30s-CST ≤8 repetitions was used to classify patients with or without limitations in mobility [24]. The cut-off value was determined based on national guidelines [26].

#### Timed up and go (TUG)

The TUG test assesses basic mobility and reflects a person’s ability to rise, walk 3 m and turn around [25]. Timeframe for administer the TUG is approximately 5–10 min. Wearing their regular footwear, participants were asked to complete the following as fast as safely possible: rise from an armchair (46 cm high), walk 3 m (marked by tape), turn, return and sit down. The use of a walking aid was allowed during the test. Limitations were determined by the use of a cut-off value > 12 s [25, 27, 28].

#### Other measurements

Demographic and clinical variables with a known association with readmission were extracted from the Danish National Patient Registry. These included age, gender and comorbidity measured with the Charlson Comorbidity Index (CCI). CCI is a weighted measure of the burden of chronic illness [29, 30]. Length of stay and marital status at baseline were used to describe the study participants.

### Statistical analysis

Means with standard deviations and percentages were calculated for subject demographic and clinical variables. Medians with the interquartile range were used for skewed data. We stratified subjects by readmission status: either yes or no. A chi-square test was used to determine if the distributions of categorical variables differed significantly between readmitted versus non-readmitted patients. T-tests (normally distributed data) and Wilcoxon rank sum tests (skewed data) were used to test for group differences on continuous variables.

Associations between limitations in performing daily activities and risk of readmission within 26 weeks were examined using odds ratios (OR) in logistic regression analysis with 95% confidence intervals (CI) as estimates. Pre-determined cut-off values for each measurement were used to examine the association between the different predictor variables and the risk of readmission. First, univariate logistic regression analyses were conducted between each measure of limitations in performing daily activities and readmission within 26 weeks.

Next, a multivariate analysis controlling for the influence of age, gender and comorbidity was conducted. The variables were selected a priori based on knowledge acquired from the literature concerning their association with the risk of readmission. Both the unadjusted and adjusted analyses were performed separately for each measurement. All tests were two-tailed, assuming a 5% significance level. Analyses were performed using STATA 15 (Stata Statistical Software, College Station, TX). The reporting of the study complies with the Strengthening the Reporting of Observational Studies in Epidemiology (STROBE) statement to ensure transparent reporting [31].

## Results

A total of 179 patients aged 65 years were invited to participate in the study of which 144 (80%) agreed and were included in this study (see flowchart in Nielsen et al. [20]). Participants mean age was 80.7 (SD = 7.9) years. Fifty-five percent of the patients were females and 39% were married. The 30s-CST was performed by 126 patients (88%), the TUG was performed by 119 patients (83%) while the AMPS was performed by 96 patients (67%). Table 1 presents the baseline characteristics for the total study sample, stratified by readmission (yes/no). Sixty-four patients (44%) were readmitted within 26 weeks after discharge. Patients who were readmitted were more likely to have a moderate to high comorbidity score than those who were not readmitted. Likewise, patients readmitted within 26 weeks had significantly lower AMPS motor and 30s-CST scores when compared to patients who were not readmitted.

**Table 1:** Baseline characteristics of the study participants (*n* = 144)

	All patients (*n* = 144)	Readmitted (*n* = 64)	No readmission (*n* = 80)	Test for difference^a^
Mean age, years (SD)	80.7 (7.9)	80.0 (7.7)	81.2 (8.1)	*p* = 0.353
Female, n (%)	79 (55)	35 (44)	44 (56)	*p* = 0.970
Married, n (%)	56 (39)	24 (43)	32 (57)	*p* = 0.523
Comorbidity, n (%)^b^				*p* = 0.002
Low: Score 0–1	75 (52)	23 (36)	52 (65)	
Moderate: Score 2–3	45 (31)	26 (41)	19 (24)	
High: Score > 4	24 (17)	15 (23)	9 (11)	
Admission diagnosis, n (%)				
Diseases of the eye and adnexa	7 (4.9)	2 (3,1)	7 (6,2)	*p* = 0.881
Diseases in the circulatory system	13 (9.0)	6 (9,4)	7 (8,8)	
Diseases in the respiratory system	6 (4.2)	4 (3,6)	2 (2,5)	
Diseases of the digestive system	8 (5.6)	4 (3,6)	4 (5,0)	
Musculoskeletal diseases	15 (10.4)	6 (9,4)	9 (11,5)	
Symptoms and abnormal clinical findings	8 (5.6)	2 (3,1)	6 (7,5)	
Injury	25 (17.4)	12 (18,8)	13 (16,3)	
Factors influencing health status	46 (31.9)	20 (31,3)	26 (32,5)	
Other diagnosis^f^	16 (11,1)	8 (12,5)	8 (10,0)	
Days of admission, median (IQR)	0.94 (0.74;1.33)	0.95 (0.72;1.35)	0.93 (0.76;1.24)	*p* = 0.709
AMPS motor, mean (SD)^c^	1.02 (0.79)	0.72 (0.79)	1.23 (0.72)	*p* = 0.001
AMPS process, mean (SD)^c^	0.93 (0.80)	0.79 (0.97)	1.03 (0.63)	*p* = 0.138
Timed Up and Go, median (IQR)^d^	11.78 (8.87;17.97)	12.231 (9.9;20.28)	10.97 (8.72;15.82)	*p* = 0.145
30s-CST, median (IQR)^e^	7 (0;10)	5.5 (0;9)	7.5 (1.5;11)	*p* = 0.056

^a^Test for difference between the group of patients being readmitted and the group of patients not being readmitted within 26 weeks

^b^Charlson Comorbidity Index

^c^AMPS motor and process score in logits, *n* = 96

^d^Timed Up and Go, *n* = 119

^e^30s-CST, *n* = 126

^f^Other diagnosis includes diagnosis with 3 or less patients

Table 2 presents the results of the logistic regression models. The crude associations between limitations in performing daily activities and the risk of readmission were positive for the AMPS motor scale (OR = 4.38 [1.36; 14.12]). When adjusting for age, gender and comorbidity score, the association between risk of readmission and AMPS motor scale remained positive (OR = 4.17 [1.18; 14.75]), while the association between risk of readmission and the 30s-CST became stronger and significant (OR = 3.36 [1.42; 7.93]). There was no association between either the AMPS process scale and the risk of readmission or between the TUG and the risk of readmission in the crude or adjusted analyses.

**Table 2:** Association of limitations in performing daily activities and risk of readmission within 26 weeks in 144 elderly patients discharged from a short-stay unit at the emergency department

	Unadjusted analysis		Adjusted analysis^a^	
	OR (95%CI)	*p*-value	OR (95%CI)	*p*-value
AMPS motor≤1.50 logit^b^	4.38 (1.36;14.12)	*p* = 0.01	4.17 (1.18;14.75)	*p* = 0.03
AMPS motor> 1.50 logit^b^	1 (ref)			
AMPS process≤1.00 logit^b^	1.51 (0.66;3.44)	*p* = 0.33	1.49 (0.59;3.76)	*p* = 0.39
AMPS process< 1.00 logit^b^	1 (ref)			
TUG ≥12 s^c^	1.22 (0.59;2.49)	*p* = 0.59	1.59 (0.69;3.71)	*p* = 0.28
TUG < 12 s^c^	1 (ref)			
30s-CST ≤ 8 times^d^	2.05 (0.99;4.24)	*p* = 0.05	3.36 (1.42;7.93)	*p* = 0.01
30s-CST > 8 times^d^	1 (ref)			

^a^AMPS adjusted for gender and comorbidity, TUG and 30s-CST adjusted for age, gender and comorbidity

^b^AMPS motor and process, *n* = 96

^c^Timed Up and Go, *n* = 120

^d^30s-CST, *n* = 126

## Discussion

In this study, we examined the association between limitations in performing daily activities and the risk of readmission within 26 weeks in a sample of 144 elderly patients discharged from a short-stay unit in the ED. We found that limitations in performing daily activities measured by the AMPS motor scale were associated with the risk of readmission within 26 weeks in both the crude and adjusted analyses, while the associations between the 30s-CST and the risk of readmission were significantly positive when adjusted for age, gender and comorbidity. The findings are consistent with the findings from other studies [3, 32–34]. In a study of Jönsson et al., the authors found that a higher degree of dependency in activities of daily living was associated with a higher risk of readmission [32]. A study of Hoyer et al. reported an independent association between activity limitations measured with the motor subscale of the Functional Independence Measure and the risk of readmission in a sample of 9405 elderly patients [3]. Limitations in performing daily activities measured by Katz Index were in a study of Giusti et al., associated with risk of readmission in a sample of elderly patients with hip fracture [33]. Likewise, in a study of Bahrmann et al., limitations in performing daily activities measured with Barthel Index were associated with risk of readmission in elderly patients admitted to the ED [34]. However, our study differs when it comes to patients’ length of hospital stay. In our study, patients were discharged directly from the ED less than 48 h after admission. The use of performance-based test to examine elderly patients’ limitations in performing daily activities may be especially relevant in an acute setting were self-report can pose some challenges in form of patients not recognising sudden limitations [17, 35].

Our findings did not show an association between either the TUG or the AMPS process scale and the risk of readmission. In contrast to our findings, a recently published study reported that a higher TUG score was associated with the risk of readmission in a sample of 1328 elderly patients [18]. However, the study differs from ours because it included a larger study population and the study patients were recruited from both acute and regular medical wards.

In the current study, we wanted to examine whether three specific performance-based tests of activity performance would be able to identify those elderly patients at an increased risk of readmission after discharge. The identification of patients with increased risk is considered to be an important component of targeting rehabilitation services to those patients likely to benefit the most. Two systematic reviews reported that risk stratification to identify patients with increased risk was beneficial in outcomes such as reducing the risk of readmission and nursing home admissions after discharge when compared with interventions without a risk stratification approach [36, 37]. However, the findings from studies using risk stratification are not consistent, and further research is needed to determine the effectiveness of such an approach.

The use of the three performance-based tests to identify patients with limitations in performing daily activities was overall feasible in our clinical setting. However, the timeframe for using all three tests were approximately 1 h, a time investment that is not easily obtained in an acute clinical practice. In addition, not all of the included patients were able to perform all three tests due to limitations in the setting, interruptions during the assessment or therapists not having the time to carry out the tests.

We included both the TUG and 30s-CST as they are simple to administer and have been validated for use with samples of older community-dwelling people [24, 27, 38]. In a study from Bruun et al. [39] the authors reported that the concurrent validity of the 30s-CST when compared with the de Morton Mobility Index was acceptable when used in a sample of 156 elderly patients in a short-stay unit in the ED. In another ED setting with 911 elderly patients, the TUG was found to be useful in identifying patients at risk of functional decline after discharge [40]. However, no studies have examined the association between performance-based measures of activity limitations and the risk of readmission. In this study, neither the TUG nor the 30s-CST was associated with the risk of readmission in the crude analyses. The results from the 30s-CST became significant when adjusting for age, gender and comorbidity. However, in an acute clinical setting, adjusting for factors affecting the results of an assessment may be problematic due to the limited timeframe and high patient flow.

The AMPS was chosen as it provides a more comprehensive picture of the quality of activity performance than measures of basic mobility. The AMPS test has been used in different studies including populations aged 65+ [41–45]. In a study of Fioravanti et al. the responsiveness of AMPS was compared with Functional Independence Measure in elderly patients at an inpatient rehabilitation unit with the result of no differences between the two tests in detecting changes [41]. A study of Norberg et al. used the AMPS to describe ADL ability in a population of elderly people with chronic heart failure [42]. In addition, AMPS has been used within populations with dementia [43] and as outcome in intervention studies [44, 45]. Although it has been widely used, this is the first study that includes the AMPS test in an acute ED setting with elderly patients. We found an association between the AMPS motor scale and readmission but no association with the process scale. This suggests that although both scales are integrated into the AMPS, the motor scale is of particular importance when identifying elderly patients at an increased risk of readmission.

We found a relatively high proportion of readmissions, with more than 44% of the elderly patients readmitted within 26 weeks. This result is consistent with the results from other studies [5, 18]. More patients in the readmission group had a higher comorbidity score than patients in the group with no readmissions. There were no differences between the groups regarding age, gender or admission diagnosis. In a systematic review from 2011 based on 12 studies, the authors reported that factors such as age and gender do not seem to be associated with the risk of readmission in contrast to comorbidity and limitations in performing daily activities [5].

### Strengths and limitations

We consider the complete follow-up on the outcome (readmission) for all patients to be a strength of the study, thereby minimising the risk of attrition bias. Information regarding the outcomes was obtained from the Danish National Patient Registry, which has a high level of completeness [46]. To account for potential confounding by gender, age and comorbidity, we adjusted for the factors in multivariate analyses, which did not change the significance of the results in relation to the AMPS or the TUG, while the result for the 30s-CST became positive and significant. The main limitations of the current study are the relatively small sample size which affects the 95% confidence intervals and thereby the uncertainty of the results. Also, that the study participants were recruited from one university hospital is considered a limitation. In addition, one-third of the patients did not perform the AMPS and an optimized implementation of the AMPS is needed.

Since patients were not included at random, but in specified time intervals on weekdays, generalisation of the results may be hampered. However, the analysis in our previously reported study showed that patients in the intervention group appeared comparable with a control group of patients admitted in afternoons and evenings in terms of their gender, diagnosis at discharge, comorbidity and marital status [18, 20].

The cut-off value of each test was determined based on recommendations from previous studies and national guidelines [21, 23, 26]. However, none of those studies included elderly patients in an acute setting, and the use of pre-specified cut-off values may have introduced the risk of misclassifying the exposure variables. Further research examining different cut-off values for the performance-based tests in larger samples in acute settings could contribute important information regarding the potential for using performance-based tests in an acute setting.

## Conclusion

Limitations in performing daily activities, measured by the AMPS motor scale and the age, gender and comorbidity adjusted 30s-CST, were associated with the risk of readmission within 26 weeks in a sample of elderly patients discharged from a short-stay unit in the ED. The results were limited by one-third of the patients did not perform the AMPS and the association between 30s-CST and risk of readmission were only positive when adjusted for age, gender and comorbidity. More studies are warranted to verify the significance of identifying and targeting patients in need of rehabilitation interventions aimed at reducing the risk of readmission.

## Data Availability

The datasets analysed during the current study are available from the corresponding author on reasonable request.

## Abbreviations

AMPS: Assessment of Motor and Process Skills
ED: Emergency Department
DSC: Dynamic Silver Code
LACE index: Length of stay, Acuity of admission, Charlson comorbidity index and Emergency department visits
TUG: Timed Up and Go
30s-CST: 30s- Chair Stand Test
